# Book Review

**Published:** 1928

**Authors:** 


### Reviews of Books

**A Short History of Medicine.** By CHARLES SINGER, M.D., D.Litt., F.R.C.P. Pp. 368. Illustrated. Oxford: Clarendon Press. 1928. Price 7s. 6d.—This is a most interesting account of modern medical discoveries and successes, showing how they have followed during the last three or four centuries from the revived investigation of the laws of the physical sciences by measurement and experiment. It is prefaced by some all too brief chapters on the development of medicine from ancient times, especially on the Greek spirit and scientific method. One of the most striking chapters is the Epilogue, which discusses whether further progress is possible, or whether the engine which has carried us so far—" the scientific method," " the determinist thought,"—is not self-limited, although so many diseases are still not curable, and those men who reach a certain age have to-day no better chance of life than they would two hundred years ago. The story of the gradual growth of physiology, pathology and public health is beautifully told, though sadly limited by considerations of space and of the readers for whom it is designed. It may be said that this brilliant study of the principles underlying the science of medicine should not be called a history of medicine, but the author replies that it is a history of ideas in this branch of biological science, and the reader should bear this in mind.

**How to Start in General Practice.** By J. G. BRIGGS, M.R.C.S. Pp. 158. London: John Murray. 1928. Price 6s. —Hospitals do not equip men for private practice, it has been said lately, as pupilage prepared our forebears, and this is another book purporting to supply their insufficiency. The author has studied the means whereby, with little capital, place and foothold may be found, or a house economically constructed and adapted to medical needs. Indeed, he is rich in device. " Have a hidden push-bell from surgery desk to maid (who can be summoned to remind you of an appointment and so cut short the visit of a too loquacious patient) ! " In weakening the veracity of the maid the author lessens our interest in her master. Already, obviously, the latter is succeeding. One so wide awake and ready for emergencies, so handy with drugs, so thrifty, so methodical with records and accounts and so faithful to his own improvement, should not miscarry in his adventure nor fail in his lot.

**Clinical Examination of the Lungs.** By E. M. BROCKBANK, M.D., F.R.C.P. and A. RAMSBOTTOM, M.D., F.R.C.P. Second Edition. Pp. viii., 112. 35 illustrations. London: H. K. Lewis & Co. Ltd. 1928. Price 5s.—This small book should prove of real value to students and medical practitioners. In a short space it summarises clearly the theory and practice of the art of physical examination of the lungs in health and disease. Most of the statements with regard to physical examination are thoroughly sound and will pass without comment. Chapter IV., however, which deals with auscultation, serves to emphasise the deplorable lack of uniformity in nomenclature which makes the teaching of students so difficult at the present time. The classification of adventitious sounds and the use of the terms râle, crepitation and rhonchus by various authors differs so widely that one is forced, when speaking of another's description, to say that it doesn't much matter what he calls things if only he knows what he means. Chapter VII., which attempts to correlate physical signs with common pulmonary disease, is necessarily very sketchy, perhaps the book would be better without it. It sets out no new matter that is not already contained in the earlier chapters. The X-Ray plates at the end of the book are excellent.

**Acute Aplastic Anæmia.** By A. H. SMITH. Pp. viii., 80. 3 figures. London: H. K. Lewis & Co. Ltd. 1928. Price 6s. —This monograph forms a case report " par excellence." Clinical observations and methods of treatment are dealt with in full detail. The results of blood examinations provide numerous points of interest, and it is a remarkable feature of the case that the erythrocytes suffered little. A few observations on the normal blood picture in children would have been a useful addition to the section on differential diagnosis. The main feature, however, is the use of liver extract in treatment, and is of particular interest at the present time, especially as little success has yet been attained in aplastic anæmia.

**Tuberculous Intoxications.** By J. HOLLOS, M.D. Pp. x., 132. Edinburgh : E. and S. Livingstone. 1928. Price 10s. 6d. —As foretold in the preface, this book "will astonish many of us, it will shock others, it will convince some." The views expressed throughout this compilation of deductions by Dr. Hollos may seem to some to illustrate how a belief, perhaps brilliantly true at its conception, may so obsess the mind of a worker, that he sees in it the explanation of widely-different phenomena. That certain individuals may be hypersensitive to tuberculous toxins, and therefore display certain reactions, is no doubt true : but to claim, as does Dr. Hollos, that amongst these reactions are such widely-different manifestations as epistaxis and rheumatic arthritis seems a conclusion wholly unjustifiable. Dr. Hollos, from the nature of his research, perhaps knowingly and defiantly lays himself open to criticism, for he claims that only in those types of abnormality which do not present the usually accepted phenomena of tuberculosis, but which he assumes really to be of this nature, do these "lytic reactions" occur ; an indulgent reader will perhaps tolerate these suggestions ; but it requires an innocent credulity to accept the further conclusion that a few drops of immune blood, rubbed lightly into the skin, is the appropriate cure for dysmenorrhœa or a ganglion of the wrist. Unhappily, in the narrations of cases, we observe a vast degree of special pleading, and a zeal for victory, more than that which ought always to be the great aim of the investigator—the discovery of truth.

**Fascial Grafting in Principle and Practice.** By H. C. ORRIN, F.R.C.S. Pp. 92. 21 plates. Edinburgh : Oliver and Boyd. 1928. Price 7s. 6d.—The employment of fascial grafts and of living sutures has become very popular of late. Many articles advocating their use in a great variety of different conditions have appeared from time to time in various medical journals. Mr. Orrin has summarised the work of others besides embodying in this small volume the results of his own experience. He also includes some very useful technical hints. Most surgeons have probably employed fascial grafting for the cure of ventral and recurrent inguinal hernias. To many, however, its use as a means of remedying adherent scars, for defects of the bladder, urethra, ureter, for fæcal fistula, or to improve the condition following a facial paralysis or ptosis, might not suggest itself. The book is a short one and easily read. It contains good photographs, a useful bibliography and a large number of split infinitives.

**Handbook of Medical Jurisprudence and Toxicology.** By W. A. BREND, M.A., M.D., B.Sc., Barrister-at-Law. Sixth Edition. Pp. xiv., 327. London: Charles Griffin & Co., Ltd. 1928. Price 10s. 6d.—At the present time when a medical curriculum is crowded with an increasing number of subjects, it is essential for teachers and students alike to make some distinction regarding the relative importance of the varied objects of study. It is obviously impossible for anyone to become an expert medical jurisconsult or a toxicologist without long and serious study, but it is not difficult to obtain a working knowledge of forensic medicine and toxicology in a short time by a judicious use of text-books. This selection is largely in the students' hands, and we are acquainted with no work which deals with the subjects referred to in a clearer way than does this small volume written by Dr. Brend. The author has spared no pains to render the work suitable for use by medical students and practitioners, it is in no sense a "cram book," but is written in such a lucid way that the reader imbibes accurate knowledge with little effort. The various sections are well up to date—as evidenced by the inclusion of recent tests for blood and references to new statutory enactments affecting medical practitioners. The convenient size and clear print contribute in no small degree to the excellence of the book, and we have no hesitation in recommending it to all those who wish to obtain a working knowledge of a very interesting subject.

**Clinical Surgery.** By J. W. DOWDEN, M.B., F.R.C.S. Pp. 68. Edinburgh: Oliver and Boyd. 1928. Price 2s.—The issue of this small volume comes as a welcome addition to the numerous text-books which a medical student is called upon to read. This is saying a good deal, nevertheless it is true. Of the sixty-eight pages there is not a dry one. The book is written in Mr. Dowden's pleasing style, and deals with the management of any surgical case from first to last. A few general hints to the beginner precede a chapter on facts which may be learnt by cultivating one's power of observation. Then follows some sound advice on the systemic examination of a patient and a guide to history taking. A few pages are devoted to certain principles of treatment, and a brief paragraph deals with prognosis. The order of case taking advised is not that universally accepted, but this is a minor point. Every student would do well to read and almost to learn the book by heart, before actually commencing hospital appointments. Such a proceeding would be of inestimable benefit to many students and patients.

**A Code of Rules for the Prevention of Communicable Diseases in Schools.** By THE MEDICAL OFFICERS OF SCHOOLS ASSOCIATION. Ninth Edition. Pp. 74. London: J. and A. Churchill. 1928. Price 2s. 6d.—The most important alterations in this edition are the more precise and detailed directions in reference to chemical disinfection, and a most excellent appendix summarising the present position in regard to the recognition of susceptibility to diphtheria and scarlet fever, and of the methods of immunisation and treatment by means of their several antitoxins. This code of rules, coming from so authoritative a source, will do much to bring about uniformity of practice in public and preparatory schools. The information provided will prove of the greatest service, not only to School Medical Officers, but to general practitioners, and all who are responsible for the health of children at school.

**Diseases of the Larynx.** By HAROLD BARWELL, F.R.C.S. Third edition. Pp. xvi., 278. Illustrated. London: Oxford University Press. 1928. Price 12s. 6d.—The third edition of this excellent little manual follows at rather a long interval after the second. The eighteen intervening years have witnessed so great a development that it has been necessary to rewrite the whole book. Nevertheless, the size has not materially increased, in spite of generous print and numerous and handsome illustrations: the latter have the great merit of being drawn by the distinguished laryngologist-author. A volume devoted to diseases of the larynx and œsophagus only, and intended for the student and practitioner rather than the specialist, is not often seen nowadays, but those in search of such a work will not find a better.

**Crawford W. Long and the Discovery of Ether Anæsthesia.** By F. L. TAYLOR, M.D. Pp. xiv., 237. 8 plates. London: Oxford University Press. 1928. Price 18s.—Mrs. Frances Long Taylor wrote this account of her father, Dr. Long's life, in order that his direct descendants might have a record of his exceptional career and valuable work. The book was intended for circulation among members of the Long family, and for this reason contains much domestic detail; but it affords us a very vivid picture of a progressive man making good in spite of adverse circumstances. We read that Dr. Long was convinced, by *post-mortem* evidence, that pulmonary tuberculosis was a curable affection, and as a result he treated phthisical patients with open air and special dieting. In surgery his technique as regards carcinoma was evidently well in advance of that of his time, for he took infinite pains when operating upon a breast to clear out axillary glands and remove soft tissues from the ribs. On the outbreak of the American Civil War, one of his first precautions was to lay in a large store of iodine for the treatment of wounds. His most important discovery occurred in 1842, when he made the first recorded use of ether as an anæsthetic during a surgical operation. In this connection his fame has now become world wide, and the justice of his claim to priority in the employment of ether anæsthesia has been established by the evidence brought forward by Mrs. Taylor, a fact which makes this book a valuable addition to the archives of medical evolution.

**The Medical Annual, 1928.** Forty-sixth year. Pp. c., 630. Illustrated. Bristol : John Wright & Sons. 1928. Price 20s. —We think that the publishers and editors have every reason to be pleased with this year's issue, for the reader will find that it contains a specially rich harvest of medical and surgical gains, many of them no doubt of minor importance, though some bid fair to be of great and permanent value. Thus the discovery of a serum which aborts poliomyelitis will be of immense value if the supply can be largely increased and really early diagnosis ensured. Possibly the latter difficulty may be met if some test can be found like the Schick reaction for diphtheria. We should like here to draw attention to the beautiful coloured plates which are given of this test, and of the scarlatina one, with the account of the results obtained. The present volume, indeed, has no less than 53 plates and 130 illustrations, some of them of high artistic merit. Among the many attractive articles we may instance the account of the liver treatment of pernicious anæmia and a useful discussion of our present knowledge of the disease ; a similar discussion on epilepsy, describing the ketogenic diet for young patients which seems to have given some cures ; a practical article on sprains and synovitis ; one on the great help given by anti-gas serum in abdominal operations, and not least, one on the management of boarding schools during epidemics with its many trying problems. There are, too, records of the discovery of an organism peculiar to trachoma ; of an antidote for cocaine poisoning ; a new treatment for burns by tannic acid ; and one for drug addiction by narcosan, a solution of lipoids, which gave striking results when used in New York on 366 patients. Readers will highly appreciate the handy directories and lists of new books, instruments and drugs which are an important feature of this most necessary book.

